# Parapharyngeal Fat Pads as an Obesity‐Related Parameter for Apnea‐Hypopnea Index Severity

**DOI:** 10.1111/jsr.70365

**Published:** 2026-05-24

**Authors:** Antonia Felix, Amro Daboul, Tatyana Ivanovska, Peter Meisel, Ralf Ewert, Ingo Fietze, Thomas Penzel, Markus Krüger

**Affiliations:** ^1^ Department of Prosthodontics, Centre for Dental, Oral and Maxillofacial Medicine University of Greifswald Greifswald Germany; ^2^ Faculty of Electrical Engineering, Media and Computer Science East Bavarian Technical University of Amberg‐Weiden Weiden Germany; ^3^ Department of Periodontology, Centre for Dental, Oral and Maxillofacial Medicine University of Greifswald Greifswald Germany; ^4^ Department for Internal Medicine B University of Greifswald Greifswald Germany; ^5^ Interdisciplinary Sleep Medicine Centre Charité University Hospital Berlin Germany

**Keywords:** apnea‐hypopnea index, body‐mass‐index, obesity, obstructive sleep apnea, parapharyngeal fat pad

## Abstract

In this study, the association between obstructive sleep apnea severity and the parapharyngeal fat pad volume is examined in a population‐based sample. We included 680 participants who underwent standardised overnight polysomnography between 2008 and 2012 as part of the Study of Health in Pomerania in a sleep laboratory in Greifswald, Germany. The apnea‐hypopnea index was used as a marker of obstructive sleep apnea severity and the parapharyngeal fat pad volume, as our variable of interest, was analysed based on Magnetic Resonance Imaging data by automated image segmentation. We performed an ordinal logistic regression analysis to test the effect of parapharyngeal fat pad volume on apnea‐hypopnea index, controlling for body‐mass index as a confounder. The results indicated that greater parapharyngeal fat volume was associated with a higher apnea–hypopnea index in an unadjusted analysis. After adjustment for body‐mass index, this association was no longer significant. An exploratory body‐mass index‐stratified analysis suggested that the association between parapharyngeal fat volume and apnea–hypopnea index may be present primarily in non‐obese individuals. These findings highlight the importance of considering body‐mass index as a measure for overall obesity when evaluating the specific role of parapharyngeal fat in obstructive sleep apnea.

## 1 Introduction

Obstructive sleep apnea (OSA) is defined as a sleep‐related breathing disorder involving repeated episodes of upper airway obstruction during sleep, which may be partial (hypopnea), complete (apnea) or a combination of both (Lee and Sundar 2021). General risk factors are male sex, age and obesity (Bonsignore 2022).

The pathogenesis is multifactorial, with contributing factors such as a narrow upper airway lumen, unstable respiratory control mechanisms, low arousal thresholds, reduced lung volumes and impaired function of upper airway dilator muscles (Bloomgarden 2023). These respiratory disturbances have been shown to contribute to the development of cardiovascular diseases including arterial hypertension and stroke. In addition, they have been demonstrated to increase the risk of diabetes, premature death and a reduction of cognitive functions as well as quality of life (Antonaglia and Passuti 2022; Vogler et al. 2023). According to the American Academy of Sleep Medicine (AASM), the apnea‐hypopnea index (AHI) is one appropriate method to categorise OSA severity (Kapur et al. 2017). This standard metric for assessing OSA is calculated as the number of apneas and hypopneas occurring during sleep divided by the total sleep time in hours, which is classified as mild (≥ 5 and < 15 events per hour), moderate (≥ 15 and < 30 events per hour) or severe (≥ 30 events per hour) (Budhiraja et al. 2019).

Obesity has long been recognised as an important risk factor for the development of OSA (Kang et al. 2014; Soylu et al. 2012; Zhou et al. 2024). The most commonly used indicator to assess obesity within the context of OSA risk is the Body Mass Index (BMI). However, BMI has its limitations, including the fact that it does not take fat distribution into account. Its accuracy is limited, particularly in patients with an increased amount of fat‐free mass (e.g., athletes) (Jabłonowska‐Lietz et al. 2017). In this regard, measuring body fat distribution could be more accurate.

There is evidence that localised fat deposits on the pharyngeal level have an impact on the collapsibility of the upper airway (Li et al. 2012). Hence, the role of fat especially in the neck region could be of interest. In the neck area, there is a distinct accumulation of adipose tissue, known as the parapharyngeal fat pads (PPFP). These are located in close proximity to the upper airway. Their shape is not predetermined and can vary significantly from person to person. In general, it can be stated that the PPFP consist of two distinct parts and can be identified by imaging techniques such as MRI or CT (Ivanovska et al. 2021b). The PPFP volume allows us to analyse the adipose tissue in the immediate proximity of the respiratory tract. Volume‐based assessments, using measurements in for example, cubic centimetres (cm^3^) could offer a superior alternative by providing direct quantification of tissue volume, avoiding the limitations of general indices like the BMI. A previous study showed that patients with lateral collapse of the pharyngeal wall had significantly larger PPFP areas than those without complete collapse (Chen et al. 2019). Another study in the same vein demonstrated that patients with OSA were found to have significantly greater pharyngeal adipose tissue indicating structural differences in the upper airway (Pahkala et al. 2014). Similarly, in a recent study it was discovered that a higher BMI was associated with larger pharyngeal soft tissues including larger fat pads in patients with OSA (Xu et al. 2025). Furthermore, in a cross‐sectional study it was observed that PPFP volume was responsible for the collapsibility of the retropalatal airway during dynamic upper airway obstruction (Jang et al. 2014). However, the fat pads were not in close proximity to the lateral pharyngeal wall to compress it. It is conceivable, that an increase in surrounding pressure of the retropalatal pharyngeal wall may have occurred due to the elevation of fat pad volume. A further study that examined the mechanical factors influencing the tendency of the pharynx to collapse in patients with OSA also concluded that it is primarily influenced by surrounding pressure (Oliven et al. 2010).

Nevertheless, there is still no consensus in the literature on whether an increase in PPFP volume contributes to the development or severity of OSA (Arens et al. 2011; Rodrigues et al. 2018; Sutherland et al. 2023; Turnbull et al. 2018).

Further investigation is needed to clarify the role of the PPFP volume in upper airway collapsibility and the pathogenesis of OSA. We hypothesise that PPFP volume may serve as a relevant anatomical parameter that is associated with OSA severity. In this study, we utilised state of the art overnight polysomnography and high‐resolution MRI data derived from a well characterised population‐based cohort in northeastern Germany. PPFP volume was quantified using an automated MRI segmentation technique and then evaluated in relation to the apnea‐hypopnea index (AHI).

## 2 Materials and Methods

### 2.1 Study Design and Population

The data which are used in the investigation were acquired in the Study of Health in Pomerania (SHIP) (Völzke et al. 2011). SHIP is a population‐based cohort study, which aims to describe health‐related conditions with the broadest possible perspective. The two main objectives are the assessment of the prevalence and incidence of common risk factors, subclinical disorders as well as the exploration of the complex relationships with the development of clinical diseases.

The first cohort (SHIP‐Start, 1997–2001) did not assess sleep‐related parameters, whereas the second cohort (SHIP‐Trend, 2008–2012) incorporated laboratory‐based polysomnography (PSG) (Fietze et al. 2019; Völzke et al. 2011). As part of SHIP‐Trend, a series of surveys and analyses were conducted, including laboratory, medical and magnetic resonance imaging data. A total of 4420 people took part in SHIP‐Trend, of which 1264 patients underwent sleep apnea polysomnography.

In the present project, complete data sets were utilised, consisting of 706 subjects with complete segmented MRI data from the SHIP‐Trend‐0 study. For the 706 participants, 680 AHI data sets were successfully obtained through the use of overnight polysomnography.

### 2.2 Sleep and MRI Data

Following the baseline assessments, which included medical evaluations, participants underwent a single‐night polysomnography (PSG) in a sleep laboratory in Greifswald, Germany (Fietze et al. 2019; Stubbe et al. 2016). The PSG complied with the 2007 guidelines established by the American Academy of Sleep Medicine (AASM) at the time of the study design (Iber et al. 2007).

Alice 5 System (Philips Respironics, Eindhoven, The Netherlands) was utilised and took place, on average, 9 days after the baseline examinations. Data collection included a comprehensive set of parameters: electroencephalogram (six channels), electrooculogram, electromyogram, electrocardiogram, nasal airflow via pressure sensor and thermistor, thoracic and abdominal movements using inductance plethysmography, body position, arterial oxygen saturation via pulse oximetry and snoring detection through a microphone. The participants were expected to sleep for a target duration of 8 h, with a minimum of 5 h required for valid data.

We utilised the AHI as our primary outcome measure (Fietze et al. 2019).

#### 2.2.1 PPFP Volume—MRI Sequence Information

For our study, we utilised MRI data from the SHIP study, which provides whole‐body imaging with detailed scans of the whole body, including head and neck scans. We adopted the previously analysed data on the MRI slice that best represents the parapharyngeal fat pads from Ivanovska et al. (2021b). From three possible MRI sequences that could be considered (head MRI, spine MRI and neck MRI), the neck sequence was chosen, which contains 40 slices for each subject and covers the entire region of interest. To analyse the parapharyngeal fat pad areas, a method of automated MRI segmentation was used (Ivanovska et al. 2021b): In brief, a fully automated algorithm was developed that extracts the fat pads without user intervention. [Correction added on 10 June 2026, after first online publication: In the preceding sentence, the citation of Ivanovska et al. has been updated from 2021a to 2021b in this version for clarity.] The segmentation of parapharyngeal fat pads was performed using a 3D CNN (namely, nnUNet) network within a selected region of interest (ROI). The resulting algorithm achieved a Dice score of ~78% on a test set consisting of 20 subjects. This was qualitatively confirmed on 500 additional subjects by experts, indicating validity for practical use. Compared to manual segmentation, the automated approach reached a Dice score within the range of intra‐observer agreement, indicating that its performance is comparable to that of an experienced human reader. Figure 1 illustrates the comparison of the automated segmentation and the segmentation performed by a human observer. This approach yielded substantial time savings, attributable to the more manageable size of the dataset and enables the efficient analysis of large epidemiological data sets to investigate the influence of adipose tissue on the development of OSA.

**FIGURE 1:**
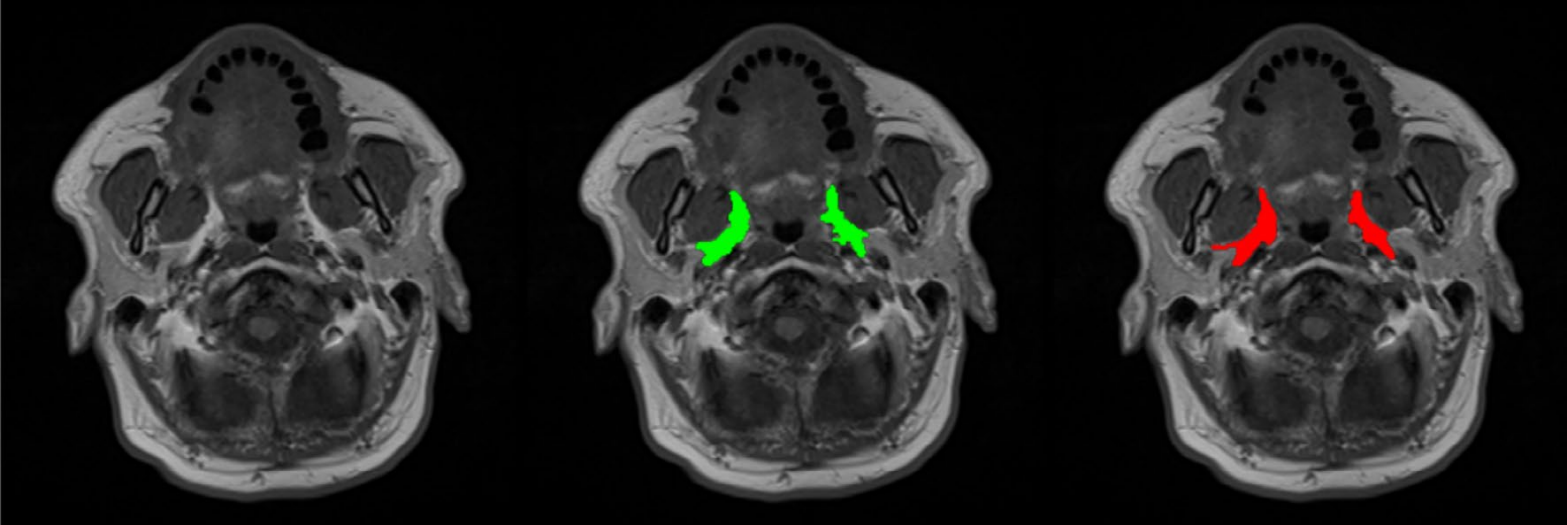
An example slice of one subject with overlaid segmentation masks. Left: The original data; Middle: Segmentation results from the human observer (green); Right: Segmentation results from the 3D nnUNet trained on z‐cropped data (red).

### 2.3 Statistical Analysis

We used the categorised apnea–hypopnea index (AHI) as the outcome variable, reflecting OSA severity. The AHI represents the number of apneas and hypopneas per hour of sleep and was classified as follows: AHI < 5: normal, 5 ≤ AHI < 15: mild, 15 ≤ AHI < 30: moderate, AHI ≥ 30: severe. This categorisation was used to account for the non‐normal distribution of AHI values.

The variable of interest PPFP volume in cm^3^ was analysed in order to evaluate its impact on the AHI. We used the ordinal logistic regression to assess the relationship between the severity of sleep apnea and calculated odds ratios (ORs).

The distribution of participants was categorised by PPFP volume and BMI with consideration given to the four AHI severity levels. BMI was utilised as a confounding variable.

R Statistics (R Core Team. 2024) with R Studio (Posit team 2025) and the packages haven (Wickham, Miller, et al. 2023), dplyr (Wickham, François, et al. 2023), MASS (Ripley et al. 2025), ggplot2 (Wickham et al. 2024), brant (Schlegel and Steenbergen 2020), car (Fox et al. 2024), ordinal (Christensen 2024) and tableone (Yoshida et al. 2022) were used for data analysis and visualisation.

## 3 Results

### 3.1 Descriptive Analysis

In Table 1 the mean PPFP volume measured in cm^3^ and the BMI are grouped according to the severity of AHI classes, are summarised. Descriptively, the mean volume of PPFP and BMI increases with the four categories of the AHI.

**TABLE 1:** PPFP volume in cm^3^ and BMI in relation to the different severity levels of AHI.

	AHI < 5: normal	5 ≤ AHI < 15: mild	15 ≤ AHI < 30: moderate	AHI ≥ 30: severe
*N* (%)	366 (53.8)	166 (24.4)	98 (14.4)	50 (7.35)
PPFP volume (cm^3^), mean (SD)	6.959 (2.642)	7.622 (2.305)	7.932 (2.489)	8.201 (2.496)
BMI (kg/m^2^), mean (SD)	26.6 (4.0)	29.3 (3.9)	30.5 (4.3)	31.0 (4.5)

*Note:* Participants (*N* = 680).

Abbreviations: AHI, apnoe‐hypopnea index; BMI, body mass index; PPFP, parapharyngeal fat pad; SD, standard deviation.

Table 2 summarises the demographic, anthropometric and polysomnographic characteristics of the study population stratified by weight status (underweight/normal weight BMI < 25 and overweight/obese BMI ≥ 25).

**TABLE 2:** Population characteristics, categorised according to weight‐status, in two groups.

Weight status	Underweight/normal weight	Overweight/obese	*p*
*N*	179	501	
Age (years), mean (SD)	45.3 (13.9)	55.1 (12.5)	< 0.001
BMI (kg/m^2^), mean (SD)	23.0 (1.50)	30.0 (3.51)	< 0.001
PPFP volume (cm^3^), mean (SD)	5.95 (2.32)	7.85 (2.46)	< 0.001
AHI, mean (SD)	4.33 (7.75)	11.6 (14.0)	< 0.001
AHI categories, *n* (%)			
AHI < 5: normal	140 (78.2)	226 (45.1)	< 0.001
5 ≤ AHI < 15: mild	27 (15.1)	139 (27.7)	
15 ≤ AHI < 30: moderate	8 (4.5)	90 (18.0)	
AHI ≥ 30: severe	4 (2.2)	46 (9.2)	
Sex, *n* (%)			
Male	77 (43)	267 (53)	0.023
Female	102 (57)	234 (47)	
TST (min), mean (SD)	391.27 (57.27)	372.91 (61.50)	0.001
Mean SpO_2_ (%), mean (SD)	96.10 (1.11)	94.95 (1.55)	< 0.001
Min SpO_2_ (%), mean (SD)	90.21 (8.24)	86.52 (7.65)	< 0.001
Snoring TST (%), mean (SD)	9.87 (13.20)	22.31 (20.62)	< 0.001

*Note:* Participants (*N* = 680); *p*‐values were calculated using ANOVA for continuous variables and *χ* ^2^ tests for categorical variables.

Abbreviations: AHI, apnoe‐hypopnea index; BMI, body mass index; PPFP, parapharyngeal fat pad; SD, standard deviation; SpO2, oxygen saturation; TST, total sleep time.

Figures 2 and 3 show boxplots with data points for the volume of PPFP and BMI sorted according to the four severity classes. The first four boxplots show that for each severity class the volume of the median PPFP is larger than the last one (Figure 2). The same is true for the BMI (Figure 3). [Correction added on 10 June 2026, after first online publication: The citations for Figures 1 and 2 in the preceding sentences have been updated to Figures 2 and 3, respectively, in this version for clarity.]

**FIGURE 2:**
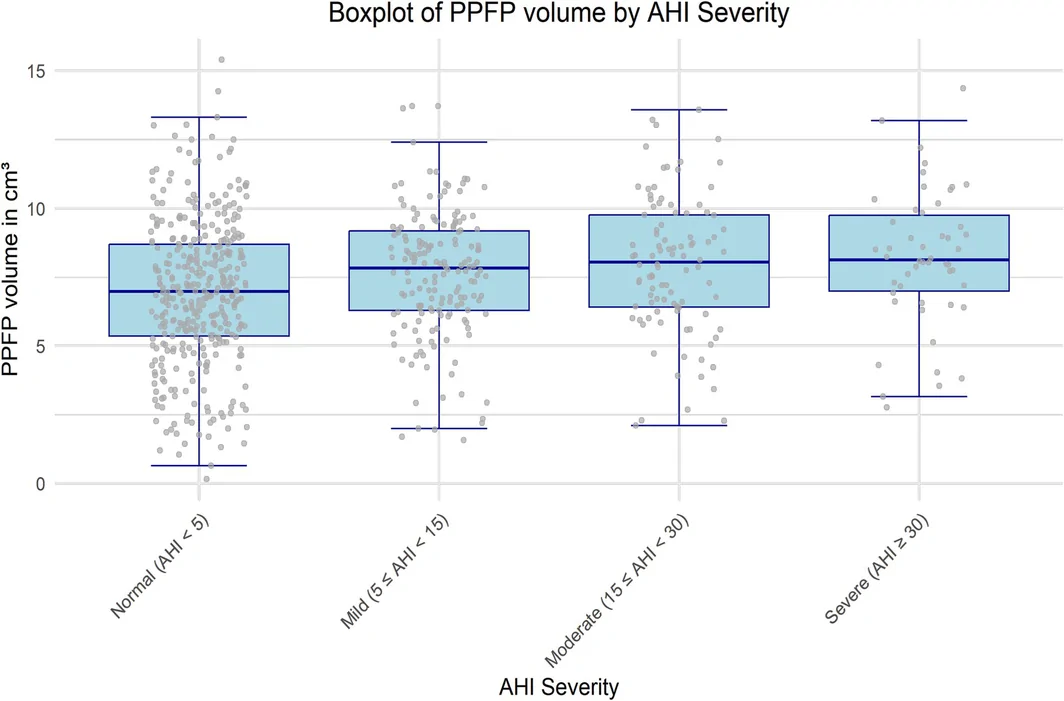
Boxplots of PPFP volume measured in the different AHI severity classes.

**FIGURE 3:**
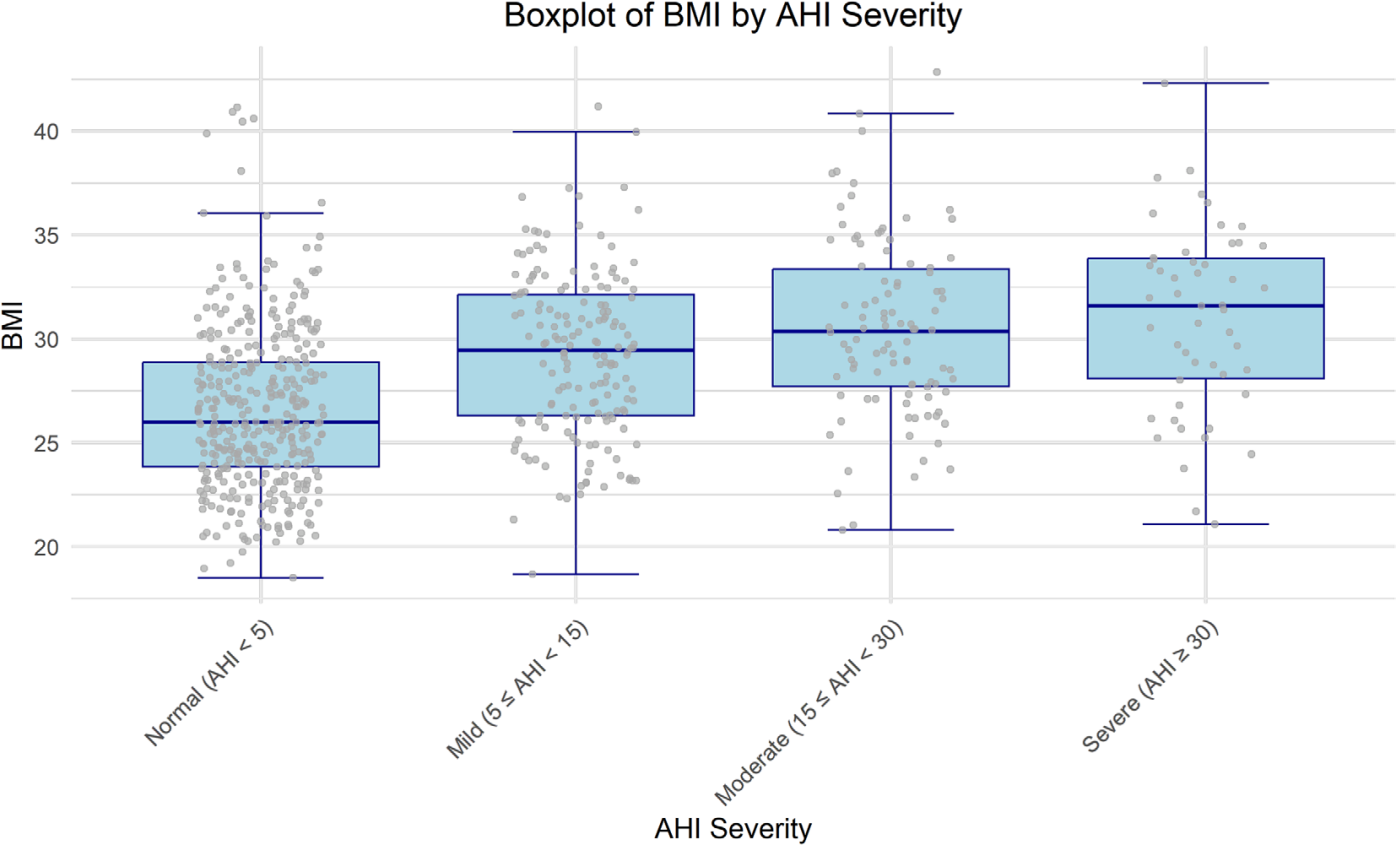
Boxplots of BMI measured in the different AHI severity classes.

### 3.2 Ordinal Logistic Regression Analysis

First, we assessed the potential impact of the PPFP volume on the AHI. To achieve this, an ordinal logistic regression model with the continuous variable of interest PPFP volume on AHI according to the four severity classes was conducted. The variable of interest PPFP volume was significantly associated with AHI, OR 1.15 (CI 1.08; 1.22), *p* < 0.001. Secondly, a control was conducted using the confounder BMI (kg/m^2^). The result no longer shows a significant association, OR 1.03 [CI 0.96; 1.10], *p* = 0.39, between the variable of interest and AHI.

For further exploration, the Pearson correlation between BMI and PPFP volume was computed. A moderate correlation, *r* = 0.39, indicated partial overlap, consistent with the assumption that both represent related but distinct facets of fat distribution. To further disentangle these findings, the adjusted ordinal logistic regression model was repeated, stratified by weight status: underweight and normal weight (BMI < 25) versus overweight and obese (BMI ≥ 25) (World Health Organization 2000). For the underweight and normal weight group, a marginally significant association was observed, OR = 1.18 [CI 1.01, 1.40], *p* = 0.052, whereas no such association was found for the overweight and obese group, OR = 1.00 [CI 0.93, 1.07], *p* = 0.95, indicating that an effect of PPFP volume on AHI might be confined to underweight and normal weight individuals (for further considerations concerning these models see Supporting Information).

## 4 Discussion

In the present study, we investigated the relationship between the PPFP volume and the severity of AHI. We found that PPFP volume was associated with AHI in the unadjusted analysis. However, this association was no longer discernable after adjusting for BMI as a potential confounder. This suggests that the apparent effect of PPFP volume on AHI may be largely explained by the BMI, indicating that PPFP volume does not independently contribute to AHI severity beyond what is accounted for by general obesity as represented by the BMI.

However, in a post hoc exploratory analysis stratified by weight status, the association persisted among underweight and normal‐weight individuals but not among those classified as overweight or obese. This pattern suggests that the PPFP volume may play an independent role in upper airway obstruction primarily among non‐obese individuals, whereas in obese patients, its contribution is likely overshadowed by the broader effects of general adiposity. One possible explanation is that in non‐obese individuals, the localised PPFP volume may exert a proportionally greater mechanical effect on lateral pharyngeal wall stability. In contrast, in obese individuals, generalised soft‐tissue enlargement and increased neck adiposity dominate upper airway dysfunction. The exploratory nature of this additional analysis limits the interpretability of this result and the finding should therefore be viewed as hypothesis‐generating rather than confirmatory.

In comparison, Chen et al. (2019) found an independent association of the PPFP area and AHI even after adjusting for BMI while Kim et al. (2022) reported that PPFP volume correlates with BMI and influences surgical outcomes. The subsequent studies also investigated the parapharyngeal fat tissue, yet in two distinctly different age groups: Carlisle et al. (2014) conducted a study on patients of advanced age where the human body develops mechanisms that serve to protect against the accumulation of fat deposits which reduces the potential for collapse. Although larger PPFP volume was associated with an increased tendency of the upper airway to collapse, the airway remained open due to the pharynx widening as a compensatory mechanism. It has been demonstrated that the structural adaptability of the upper airway, in addition to the amount of PPFP volume, is a determining factor in the development of OSA. This suggests a potential mechanism that could explain why some older men do not develop OSA despite larger fat deposits in the parapharyngeal area. On the other hand, Arens et al. (2011) undertook a study of obese children. Here, it was observed that although obese children had larger PPFP volume in comparison with obese children without OSA, no association was found with the severity of OSA. Rather, the obese OSA group had larger adenoids, tonsils and retropharyngeal nodes and there was a positive correlation with the severity of OSA. A similar conclusion was reached by Schwab et al. (2015) demonstrating that particularly an enlargement of the pharyngeal lymphoid tissue was observed in young patients with OSA, thus indicating that adenotonsillectomy could be a subsequent therapy. In their review Zaffanello et al. (2025) also concluded that adenotonsillar hypertrophy is still the most important factor in childhood OSA.

Turnbull et al. (2018) reported that increased tongue and soft palate volumes are associated with greater OSA severity in individuals with severe obesity. Notably, Chen et al. (2019) found an association between PPFP volume and lateral wall collapse only at the subglosso‐supraglottic level, with no correlation at the nasopharyngeal or oropharyngeal levels. At those levels, collapse was more linked to soft tissue structures such as the tonsils and tongue.

The anatomical interpretation of our results should be viewed within the broader context of oropharyngeal morphology. OSA is developed from the combined influence of these several interrelated structures, including the pharynx, tongue and soft palate, whose relative dimensions and spatial relationships determine airway stability. Evidence from our complementary work on upper airway morphology within the same cohort (Ivanovska et al. 2021a) also indicates that variations in the overall configuration of these oropharyngeal structures appear to contribute more consistently to AHI severity. This suggests that structural elements such as the tongue and soft palate may play more immediate roles in determining airway patency. Consequently, the PPFP volume should be regarded as part of a complex anatomical network, in which fat distribution may modulate but not solely determine upper airway obstruction.

From a methodological perspective, our analysis relied on static MRI like many previous studies on PPFP volume. While MRI provides high‐resolution anatomical data, it captures the airway in a single state and does not reflect dynamic changes during sleep. This approach was also used by the majority of studies analysing the upper airways (Li et al. 2012; Pahkala et al. 2014). Conversely, dynamic techniques such as drug‐induced sleep endoscopy (DISE) with sedatives, such as propofol, as utilised in other studies enable direct visualisation of airway collapse under sleep‐like conditions (Chen et al. 2019; Jang et al. 2014). This is also emphasised by Hong et al. (2013), where changes were evaluated in upper airway collapse according to the depth of sedation during DISE. They demonstrated that the degree of upper airway narrowing increased with greater sedation depth at the retropalatal and retroglossal level. Future studies could benefit from combining volumetric MRI data with a DISE‐based evaluation of collapse patterns. This combination could provide a more comprehensive understanding of how parapharyngeal fat contributes to obstruction. Due to the absence of consistent procedural guidelines, the integration of standardised DISE protocols and concurrent imaging could help bridge the gap between anatomical and functional assessment of the upper airway in OSA (Li et al. 2023).

Several limitations of our study should be considered. PPFP volume was analysed as a single structure without distinguishing between its separate anatomical components. This likely reduced the sensitivity of our measurements and could partly account for the absence of an independent association with AHI. More detailed, region‐specific information (i.e., measuring each fat pad separately) and higher resolution imaging might have yielded different results though it is unlikely that this alone would have substantially changed the observed association with BMI.

On the other hand, a strength of our study is its use of data from a population‐based sample of 680 participants, offering a broader representation than smaller, clinic‐based cohorts. Other studies have been limited to narrow OSA severity groups or specific patient populations (Jang et al. 2014; Kim et al. 2022; Pahkala et al. 2014). In contrast, our cohort included the full spectrum of AHI severity, assessed using the gold standard overnight polysomnography. MRI was used to assess parapharyngeal fat pad volume, offering high‐resolution, non‐invasive imaging.

Since the increased volume of parapharyngeal fat pads is still closely associated with obesity, the question remains as to how obesity, one of the most important risk factors for the development of OSA, contributes to airway collapse. As mentioned in the Introduction, some studies suggest that the surrounding pressure caused by the increased parapharyngeal fat plays an important role. In addition, other studies have examined the impact of obesity on pharyngeal function (Esmaeili et al. 2025). Tsai et al. (2009) described that an accumulation of adipose tissue in the neck region exerts a gravitational force on the airway, particularly when the patient is in the supine position. In addition, there is evidence that cytokines such as TNF‐a, as systemic inflammatory mediators associated with obesity, may also affect the mechanical control mechanisms of the pharynx (Pahkala et al. 2014). These control mechanisms mediate the collapsibility of the pharynx and therefore cytokines may increase the likelihood of developing OSA (Arias et al. 2008; Schwartz et al. 2010). Sahlman et al. (2012) showed that a sensitive marker of the proinflammatory state decreased with weight loss in patients with mild OSA. Therefore, it appears that local adipose tissue, such as the PPFP, in conjunction with general obesity, through mechanisms such as systemic inflammation, may increase OSA vulnerability.

Despite the apparent absence of an association between the size of PPFP volume and the severity of OSA in our study when adjusted for BMI, the adipose tissue may still play an indirect role in the pathophysiology. Clinically, the moderate correlation between BMI and PPFP volume suggests that while higher overall adiposity contributes to parapharyngeal fat accumulation, PPFP volume may also represent an at least partly independent regional fat depot with potential relevance for upper airway collapsibility. This implies that even among individuals with similar BMI, individual differences in parapharyngeal fat distribution could occur and influence the severity of OSA. It can be hypothesised that adipose tissue may also serve as a marker for other pathophysiological processes, such as local inflammation, which in turn influences the development of OSA. However, these hypotheses require further testing in future studies, ideally incorporating longitudinal data.

## 5 Conclusion

In the present study an association of the parapharyngeal fat pads with AHI severity is found in an unadjusted analysis only, indicating that parapharyngeal fat volume does not explain variance in AHI beyond what is already captured by general obesity. Nevertheless, in a further exploratory analysis, the association between PPFP volume and AHI appeared to be more pronounced among individuals classified as underweight or normal weight, whereas no such relationship was observed in the overweight and obese group. While this pattern raises the possibility that PPFP volume could play an independent role in upper airway obstruction among non‐obese patients, the exploratory nature of this analysis limits the interpretability of this finding. Future studies, ideally using detailed anatomical characterisation and dynamic functional imaging, are needed to clarify the contribution of parapharyngeal fat to the pathogenesis of OSA and to determine whether it provides explanatory value beyond general adiposity.

## Author Contributions

**Antonia Felix:** conceived the study, conceived and designed the analysis, wrote the paper. **Amro Daboul:** conceived the study, conceived and desgined the analysis, revised the paper. **Tatyana Ivanovska:** revised the paper. **Peter Meisel:** revised the paper. **Ralf Ewert:** revised the paper. **Ingo Fietze:** revised the paper. **Thomas Penzel:** revised the paper. **Markus Krüger:** conceived the study, conceived and designed the analysis, performed the analysis, revised the paper.

## Funding

The authors have nothing to report.

## Ethics Statement

This retrospective data analysis is based on data from the Study of Health in Pomerania (SHIP) cohort study. The study followed the recommendations of the Declaration of Helsinki. The medical ethics committee of the University of Greifswald approved the study protocol and oral and written informed consents were obtained from each of the study participants.

## Conflicts of Interest

The authors declare no conflicts of interest.

## Supporting information


**Table A1.** Associations between AHI and PPFP volume after adjustment for relevant covariables.
**Table A2**. Pairwise correlations between PPFP volume and AHI, BMI, age and sex.

## Data Availability

The data from the SHIP study are not publicly available due to restrictions in the participants' informed consent. However, access can be granted to qualified researchers upon request. Applications for data use can be submitted via the official data access portal at https://fvcm.med.uni‐greifswald.de/, provided the criteria for handling confidential data are met.
