# Supplementary material for: Parapharyngeal Fat Pads as an Obesity‐Related Parameter for Apnea‐Hypopnea Index Severity
Source: J Sleep Res. 2026 May 24;35(5):e70365. doi: 10.1111/jsr.70365 (PMC13619032; doi:10.1111/jsr.70365)
Supplement: Supplementary file 1 [file JSR-35-e70365-s001.docx]

## Supporting Information

Conversely, considering the confounder BMI, an OR of 1.14 [CI 0.89, 1.49], *p* = .32 was observed for the underweight and normal weight group and an OR of 1.18 [CI 1.12, 1.24], p < .001 for the overweight and obese group. It should be noted that the interpretation of the confounder BMI is limited, since effect estimates for adjustment variables derived from the same regression model should be interpreted with caution and are not directly comparable to the effect estimate of the primary exposure (Westreich & Greenland, 2013). Nevertheless, these results are in line with the PPFP volume being decisive in the lower weight group but not in the higher weight group.

To evaluate the robustness of the association between PPFP volume and AHI severity, additional ordinal logistic regression models were estimated adjusting sequentially for individual covariates (see Table A1, see Table A2 for pairwise correlations with PPFP volume). These are not intended to be interpreted as independent effects (Rothman, Greenland, & Lash, 2008). Results are in line with the main analysis indicating a rather weak or unstable effect of PPFP volume on AHI.

*Table A1.* Associations between AHI and PPFP volume after adjustment for relevant covariables.

|  | **underweight and normal weight** | | **overweight and obese** | |
| --- | --- | --- | --- | --- |
| **Covariable** | **OR [CI]** | **p** | **OR [CI]** | **p** |
| Sex | 1.24 [CI 1.06, 1.46] | *p* = .007 | 1.04 [CI 0.98, 1.12] | *p* = .16 |
| Age | 1.01 [CI 0.83, 1.22] | *p* = .95 | 1.02 [CI 0.95, 1.09] | *p* = .64 |
| BMI | 1.18 [CI 1.01, 1.40] | *p = .052* | 1.00 [CI 0.93, 1.07] | *p = .95* |

*Table A2.* Pairwise correlations between PPFP volume and AHI, BMI, age, and sex

| **Variable pair** | **Measure** | **r** | **p** |
| --- | --- | --- | --- |
| PPFP – AHI | Pearson r | .16 | < .001 |
| PPFP – BMI | Pearson r | .39 | < .001 |
| PPFP – Age | Pearson r | .32 | < .001 |
| PPFP – Sex | point-biserial r | -.02 | .48 |

## References

Rothman, K. J., Greenland, S., & Lash, T. L. (2008). *Modern epidemiology* (3rd Edition). Wolters Kluwer Health/Lippincott Williams & Wilkins.

Westreich, D., & Greenland, S. (2013). The Table 2 Fallacy: Presenting and Interpreting Confounder and Modifier Coefficients. *American Journal of Epidemiology*, *177*(4), 292–298. https://doi.org/10.1093/aje/kws412
